# Editorial: Stem cell therapy for hereditary neuromuscular diseases

**DOI:** 10.3389/fcell.2025.1741683

**Published:** 2025-11-19

**Authors:** Katsumasa Goto, Atsushi Asakura

**Affiliations:** 1 Department of Physiology, Graduate School of Health Sciences, Toyohashi SOZO University, Toyohashi, Aichi, Japan; 2 Stem Cell Institute, University of Minnesota Medical School, Minneapolis, MN, United States; 3 Greg Marzolf Jr. Muscular Dystrophy Center and Department of Neurology, University of Minnesota Medical School, Minneapolis, MN, United States

**Keywords:** muscle stem cell, skeletal muscle, duchenne muscular dystrophy (DMD), live imaging, cell therapy, multiple sclerosis (MS), spatial transcriptomics, muscle regeneration

Skeletal muscle cells are widespread across a range of species, from simpler organisms such as cnidarians to higher vertebrates. They represent the most considerable tissue in the body by mass and exhibit an extraordinary potential for regeneration. Skeletal muscle contains a stem cell population termed satellite cells, which serve as the progenitor cells of skeletal muscle and play a crucial role in this regenerative process (Asakura et al., 2002). Satellite cells exhibit differentiation potential toward adipocytes, osteocytes, fibroblasts, and other cell types *in vitro* or under specific pathological conditions (Asakura et al., 2001). However, the primary fate of differentiation in normal muscle regeneration is skeletal muscle cells. The discovery of satellite cells in the skeletal muscle of rats and frogs was first reported in 1961 by electron microscopy, in which they were found encircling adult muscle fibers (Mauro, 1961). These single-nucleus satellite cells reside in a dormant state outside the plasma membrane of multinucleated muscle fibers and underneath the basal lamina. When triggered by stimuli such as exercise or injury, these quiescent satellite cells activate and begin dividing into myoblasts. Myoblasts proliferate by dividing, then exit the cell cycle, begin to express genes for skeletal muscle structural proteins, and aid in the creation, repair, and growth of new muscle fibers by fusing with one another or with pre-existing muscle fibers. During this phase, some myoblasts return to satellite cells, thereby preserving the regenerative potential of skeletal muscle throughout a lifetime (self-renewal capability) (Verma et al., 2018; Hung et al., 2023). The integrity, volume, and functionality of human skeletal muscle remain intact until approximately the age of 50. However, the aging process gradually results in a reduction of muscle mass, atrophy of muscle fibers, alterations in muscle structure (including fatty infiltration and fibrosis), and diminished muscle function (Majchrzak et al., 2024; Libergoli and Almada, 2025). Nonetheless, it is feasible to postpone the aging of skeletal muscle through an energetic, health-conscious lifestyle and a well-balanced nutritional regimen. The remarkable ability of skeletal muscle tissue to recover from injury is largely attributed to satellite cells, which allow it to repeatedly undergo cycles of necrosis and regeneration while maintaining functionality and structural integrity. However, various conditions such as aging, muscular dystrophies, cancer cachexia, and diabetes can hinder the efficiency of satellite cells, failing to regenerate. Approaches aimed at boosting satellite cell function may help preserve or rejuvenate skeletal muscle mass throughout aging and in various diseases.

The regulation of satellite cell functions is governed by a dual approach that includes both internal and external factors. The microenvironment of stem cells influences the external component, referred to as the stem cell niche (Verma et al., 2018; Hung et al., 2023). To ensure the maintenance of satellite cells throughout their lifespan, various niche cell types coordinate in a spatiotemporally precise manner, regulating quiescence maintenance, self-renewal, and the trajectory of terminal differentiation. Satellite cells are situated within a complex three-dimensional (3-D) environment that includes multiple adjacent niche cells, which are essential for proper cellular function. These neighboring cells, such as mesenchymal progenitor cells, fibroblast-adipocyte progenitor cells (FAP), macrophages, eosinophils, T cells, vascular endothelial cells, and perivascular cells, are acknowledged as pivotal regulators of satellite cells and regenerative processes (Verma et al., 2018; Hicks et al., 2023; Majchrzak et al., 2024). Signals from niche cells that govern control include the extracellular matrix (ECM), growth factors, and interactions between cells (Schüler et al., 2022; Chrysostomou and Mourikis, 2024; Helzer et al., 2024). Therefore, it is crucial to understand how aging and disease affect the stem cell niche and to examine the development of therapies based on stem cells.

Inherited neuromuscular disorders comprise a diverse array of genetic conditions that affect motor neurons, peripheral nerves, skeletal muscles, or the neuromuscular junction. These disorders are major factors contributing to mortality, disability, and mental health Research Topic globally, rendering them a significant public health challenge. One notable example is Duchenne muscular dystrophy (DMD), the most prevalent type of muscular dystrophy, which is a severe X-linked disorder marked by mutations in the dystrophin gene. These genetic alterations lead to the creation of defective dystrophin-related glycoprotein complexes (DGCs) within satellite cells and skeletal muscle fibers (Robertson et al., 2024; Łoboda and Dulak, 2025). The lack of functional dystrophin causes improper muscle regeneration and heightens the myofibers’ vulnerability to injury, enhancing the disease’s progressive nature. Eventually, satellite cells reach a tipping point at which they can no longer keep pace with the degeneration of muscle fibers in dystrophic muscles, thereby advancing the disease. Progress in genetic studies since the discovery of the DMD gene in 1986 has revealed countless disease-causing mutations (Monaco et al., 1986); however, effective therapies are still scarce. Thus, the emergence of new treatments, especially those focusing on stem cell-based regenerative medicine, is of paramount importance.

This Research Topic seeks to shed light on the most recent breakthroughs in treatment strategies for hereditary neuromuscular disorders, focusing on muscle stem cell therapy and other novel therapeutic approaches relevant to conditions such as DMD. This Research Topic delved into the biological mechanisms underlying these disorders, the clinical applications of regenerative medicine, and the future opportunities offered by innovative therapies. Furthermore, this Research Topic investigated intricate multicellular interactions within muscle tissues by employing advanced methodologies, including high-resolution imaging, systems biology, proteomics, and epigenetics.

Four original review articles were published on this Research Topic. Spatial Transcriptomics has emerged as a revolutionary technology poised to reshape and accelerate our understanding of cellular communication. This aspect is particularly significant in skeletal muscle, where syncytial myofibers exist alongside a diverse array of interstitial cell populations. In the first manuscript, Virtanen et al. investigated the different types of Spatial Transcriptomics methods, provided a concise overview of available analytical tools, and emphasized recent progress in the skeletal muscle field enabled by Spatial Transcriptomics. Stem cell therapy for DMD is a promising approach to promote muscle regeneration, though the conditions for transplantation and the pre-treatment of various cell types remain under refinement. The second manuscript, by Tominari et al., outlined the obstacles to effective muscle tissue regeneration in DMD and examined the most recent developments in stem cell-based therapy for the condition. They summarized strategies for preconditioning cells for replacement therapies and for treating the disease niche to enhance muscle fiber integration.

Multiple sclerosis (MS), a persistent autoimmune disorder affecting the central nervous system (CNS) and skeletal muscle, is marked by inflammation, demyelination, and neurodegeneration, resulting in a variety of clinical manifestations such as fatigue, sensory deficits, and cognitive impairment. The third manuscript, by Sheikhi et al., provided a comprehensive analysis of mesenchymal stem cells (MSCs) for cell therapy in MS, examining mechanisms of action encompassing immune system regulation, remyelination facilitation, and neuroregeneration. Particular emphasis was placed on challenges such as delivery methods, dosing protocols, and the integration of MSCs with traditional treatments.

Recent technological advancements in imaging, such as confocal, intravital, and multiphoton microscopy, offer promising avenues for observing and delineating the dynamic morphology and behavior of satellite cells. The fourth manuscript, by Karthikeyan and Asakura, summarized live and 3-D imaging techniques that have advanced understanding of satellite cell activities, morphological alterations, interactions within the muscle microenvironment, and internal signaling networks during the transition from quiescence to activation. By merging cutting-edge imaging technologies with computational approaches, researchers can explore complex biological processes underlying skeletal muscle regeneration and associated degenerative conditions such as sarcopenia and DMD.

This Research Topic of four articles showcases the exceptional caliber of the writings featured in the Research Topic of the Stem Cell Research segment of Frontiers in Cell and Developmental Biology. It unveils a research domain advancing at an impressive rate.

## References

[B1] Asakura A. Komaki M. Rudnicki M. (2001). Muscle satellite cells are multipotential stem cells that exhibit myogenic, osteogenic, and adipogenic differentiation. Differentiation 68 (4-5), 245–253. 10.1046/j.1432-0436.2001.680412.x 11776477

[B2] Asakura A. Seale P. Girgis-Gabardo A. Rudnicki M. A. (2002). Myogenic specification of side population cells in skeletal muscle. J. Cell. Biol. 159 (1), 123–134. 10.1083/jcb.200202092 12379804 PMC2173497

[B3] Chrysostomou E. Mourikis P. (2024). The extracellular matrix niche of muscle stem cells. Curr. Top. Dev. Biol. 158, 123–150. 10.1016/bs.ctdb.2024.01.021 38670702

[B4] Helzer D. Kannan P. Reynolds J. C. Gibbs D. E. Crosbie R. H. (2024). Role of microenvironment on muscle stem cell function in health, adaptation, and disease. Curr. Top. Dev. Biol. 158, 179–201. 10.1016/bs.ctdb.2024.02.002 38670705

[B5] Hicks M. R. Saleh K. K. Clock B. Gibbs D. E. Yang M. Younesi S. (2023). Regenerating human skeletal muscle forms an emerging niche *in vivo* to support PAX7 cells. Nat. Cell. Biol. 25 (12), 1758–1773. 10.1038/s41556-023-01271-0 37919520 PMC10709143

[B6] Hung M. Lo H. F. Jones G. E. L. Krauss R. S. (2023). The muscle stem cell niche at a glance. J. Cell. Sci. 136 (24), jcs261200. 10.1242/jcs.261200 38149870 PMC10785660

[B7] Libergoli M. Almada A. E. (2025). Stem cell aging and rejuvenation in the skeletal muscle system. Rejuvenation Res. 28 (4), 158–171. 10.1089/rej.2025.0028 40556617 PMC12315999

[B8] Łoboda A. Dulak J. (2025). Cell therapy for Duchenne muscular dystrophy: promises, challenges, and controversies. Cell. Mol. Life Sci. 82 (1), 356. 10.1007/s00018-025-05904-5 41074937 PMC12515165

[B9] Majchrzak K. Hentschel E. Hönzke K. Geithe C. von Maltzahn J. (2024). We need to talk-how muscle stem cells communicate. Front. Cell. Dev. Biol. 12, 1378548. 10.3389/fcell.2024.1378548 39050890 PMC11266305

[B10] Mauro A. (1961). Satellite cell of skeletal muscle fibers. J. Biophys. Biochem. Cytol. 9 (2), 493–495. 10.1083/jcb.9.2.493 13768451 PMC2225012

[B11] Monaco A. P. Neve R. L. Colletti-Feener C. Bertelson C. J. Kurnit D. M. Kunkel L. M. (1986). Isolation of candidate cDNAs for portions of the Duchenne muscular dystrophy gene. Nature 323 (6089), 646–650. 10.1038/323646a0 3773991

[B12] Robertson R. Li S. Filippelli R. L. Chang N. C. (2024). Muscle stem cell dysfunction in rhabdomyosarcoma and muscular dystrophy. Curr. Top. Dev. Biol. 158, 83–121. 10.1016/bs.ctdb.2024.01.019 38670717

[B13] Schüler S. C. Liu Y. Dumontier S. Grandbois M. Le Moal E. Cornelison D. (2022). Extracellular matrix: brick and mortar in the skeletal muscle stem cell niche. Front. Cell. Dev. Biol. 10, 1056523. 10.3389/fcell.2022.1056523 36523505 PMC9745096

[B14] Verma M. Asakura Y. Murakonda B. S. R. Pengo T. Latroche C. Chazaud B. (2018). Muscle satellite cell cross-talk with a vascular niche maintains quiescence *via* VEGF and notch signaling. Cell. Stem Cell. 23 (4), 530–543.e9. 10.1016/j.stem.2018.09.007 30290177 PMC6178221

